# Laparoscopic Ovariectomy in a Domestic Yak

**DOI:** 10.1155/2020/8886670

**Published:** 2020-11-06

**Authors:** Drew W. Koch, Katharine M. Simpson, Jeremiah T. Easley, Eileen S. Hackett

**Affiliations:** Department of Clinical Sciences, College of Veterinary Medicine and Biomedical Sciences, Colorado State University, Fort Collins, CO 80523, USA

## Abstract

Owners of a juvenile domestic yak elected bilateral ovariectomy to prevent future reproduction. The yak was noted to be healthy at presentation. Both ovaries were removed using a laparoscopic approach as follows: after induction and maintenance of general inhalant anesthesia, 15 degrees Trendelenburg positioning was required to view the ovaries. Ovariectomy was conducted within a surgical time of 50 minutes. Due to the small ovarian size, portal enlargement was not necessary for removal. Mild hemorrhage from the left ovarian pedicle was controlled with application of a vessel-sealing device. Postoperative complications were not encountered during hospitalization. At 12 months following surgery, the yak was healthy, and the owner was highly satisfied with the procedure. The described approach was successful for performing laparoscopic ovariectomy in a juvenile yak. Positioning for surgery was similar to other small ruminant species. Further case enrollment is needed to optimize the surgical approach and better describe clinical outcomes.

## 1. Introduction

Pet owners that adopt farm animals often pursue animal care that is not dictated by production costs, in contrast to many livestock purveyors. Their decision to seek minimally invasive surgical alternatives might, in part, be informed by experiences with other pets. Laparoscopic surgical procedures are considered to be less painful and associated with shorter convalescence times. If feasible, these procedures have multiple perceived advantages when applied to pet livestock.

## 2. Case Description

A 6-month-old intact female domestic yak (*Bos grunniens*) presented for elective, bilateral laparoscopic ovariectomy due to concern about poor breeding soundness. The yak was intended to be housed with an intact male yak, and the owner wished to pursue ovariectomy to prevent pregnancy from unintentional breeding.

The yak weighed 80 kg upon presentation. During hospitalization, the yak was bottle fed with milk and was fasted for 24 hours preceding surgery. Preoperative point of care blood work was unremarkable (PCV 38%, total protein 7.0 g/dl).

Prior to surgery, a catheter was placed in the left jugular vein. Flunixin meglumine (Banamine, 1.1 mg/kg IV) and ceftiofur hydrochloride (Excenel, 2.2 mg/kg SQ) were administered. Approximately one hour prior to induction, the animal received morphine (0.05 mg/kg) and midazolam (0.05 mg/kg) each IV and IM (8 mg total of each). Induction was performed with ketamine (5 mg/kg IV), midazolam (0.2 mg/kg IV), and morphine (0.1 mg/kg IV). The yak was maintained on isoflurane inhalant anesthesia in oxygen (3 l/min) and received a maintenance rate (2.5 ml/kg/hr IV) of balanced polyionic fluids during surgery. While in lateral recumbency, an epidural was performed at the lumbosacral space (morphine 8 mg, lidocaine 24 mg, and 0.9% sodium chloride 2 ml). Anesthetic monitoring included ECG, pulse oximetry, capnography, arterial blood gas analyses, and both direct and indirect blood pressure measurements. Intermittent positive pressure ventilation was maintained throughout surgery. Then, the yak was positioned in dorsal recumbency. The ventral abdomen was clipped free of hair from xiphoid process to pubis and lateral to the flank. Aseptic surgical site preparation (alternating scrubs of 7.5% povidone-iodine solution and alcohol) was performed prior to draping.

The animal was routinely draped and portal sites were prepared similar to a previous report [1]. Briefly, a 1 cm incision was made through the skin and linea alba 1 cm caudal to the umbilicus using a #10 scalpel blade. A 12-gauge, 6.4 cm long teat cannula was placed through the incision into the abdomen, which was insufflated with carbon dioxide at a rate of 12 l/min with a commercial insufflator until a maximum pressure of 10 mm Hg was achieved. The teat cannula was removed, and a positive profile-threaded cannula (Ternamian EndoTIP 31103T4, Karl Storz Veterinary Endoscopy, Goleta, CA) without an obturator was introduced into the abdomen by controlled rotation under visual control via a 30° rigid laparoscopic telescope (Hopkins II Telescope 26003BA, Karl Storz Veterinary Endoscopy, Goleta, CA) connected to a viewing tower with a camera and light source. Two instrument portals were created approximately 10 cm caudolateral to the first portal in the right and left parainguinal region (i.e., approx. 7 cm caudal and 7 cm lateral to the initial telescope portal). For each of those 2 portals, a scalpel was used to create a 1 cm incision in the skin, and a positive profile-threaded cannula was inserted through the abdominal musculature and peritoneum and into the abdomen with visual guidance provided intra-abdominally by the laparoscopic telescope. The surgeon stood on the right side of the yak and the assistant surgeon stood on the left side of the yak throughout the procedure.

**Figure 1:**
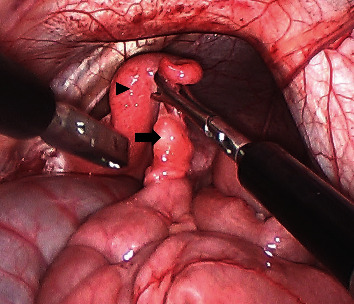
After insufflation, the uterine body was identified with the help of traumatic and Babcock laparoscopic forceps. The right uterine horn (black arrowhead) was grasped with Babcock forceps. The right ovary is visible (indicated with the black arrow). Ovaries were approximately 8 mm in diameter.

After the portals were created, the yak was tilted into the Trendelenburg position until the reproductive tract was visible (approximately 15 degrees). Traumatic laparoscopic forceps were inserted through the left instrument portal and used to grasp the left ovary. The mesovarium and mesosalpinx were identified and transected with a blunt-tip vessel sealer and divider device (Ligasure, Covidien-Medtronic, Mansfield, MA) inserted through the right instrument portal. Due to the small size of ovarian tissue (approx. 8 mm in diameter), once transected, the ovary was immediately removed through the cannula with the traumatic laparoscopic forceps. A similar approach was performed for the right ovary (Figure 1). Following ovariectomy, the ovarian pedicles were observed for hemostasis. Persistent, mild hemorrhage from the left ovarian pedicle was controlled with repeat application of the vessel sealer and divider device. The Trendelenburg positioning was then discontinued, and the laparoscopic telescope and cannulas were removed. The linea alba and external muscular fascia were apposed with a 0 synthetic glycomer 631 absorbable monofilament suture in a cruciate pattern. The skin incisions were closed with a 2-0 synthetic glycomer 631 absorbable monofilament in a Ford interlocking pattern.

The yak was maintained on supplemental oxygen during anesthetic recovery in sternal recovery, and this was discontinued when the swallow reflex returned, at which time the orotracheal tube was removed and she was returned to her stall. The yak was maintained in the hospital for observation for 48 hours following surgery, and antibiotic therapy (ceftiofur hydrochloride, Excenel, 2.2 mg/kg SQ once daily) was continued during this time. No further anti-inflammatory medications were administered. Minimal swelling of the surgical incisions was observed during this time, and the yak did not display signs of discomfort. At discharge, the owner was instructed to confine the yak in a small pen for 7 days prior to resuming pasture turnout.

The owner was contacted by phone 12 months following surgery. The yak's owner reported that mild swelling was visible at the midline sutured portal site shortly after hospital discharge and that this swelling resolved without treatment. The yak remained comfortable, returned to the herd, and did not develop further complications. The owner was very satisfied with the procedure and would recommend it for other yaks requiring ovariectomy.

## 3. Discussion

This is the first report of bilateral ovariectomy in a yak in dorsal recumbency under general inhalant anesthesia. Furthermore, this procedure was performed in a juvenile animal utilizing a minimally invasive approach. Three portals, an assistant surgeon, and a table capable of Trendelenburg positioning were required to perform the surgery. The animal recovered uneventfully after surgery, and at owner follow-up 12 months later, the animal was healthy and had returned to its herd without ongoing complications.

Multiple reports have been published examining various laparoscopic approaches in cattle for abomasopexy, reproductive monitoring, and oocyte aspiration [2–5]. Only two have focused on laparoscopic ovariectomy in the cow; however, both reported their respective techniques were feasible [6, 7]. Due to the torturous nature of the cervix in small ruminants, laparoscopic insemination, oocyte collection, and embryo transfer have been performed in those animals and, therefore, could be applicable to yaks [8]. Because no current literature exists relating to yak ovariectomy, these reports were examined, along with previous work in small ruminants, as basis for an approach to laparoscopic ovariectomy in the present animal [1]. Ovariectomy was found to be similarly easy to perform with good visibility of the caudal abdomen after combined insufflation and Trendelenburg positioning. The present report suggests that this technique could be translated to other cattle species for ovarian removal if required, avoiding large flank incisions, chain loop ecraseurs, and need for hand assistance [9–11].

Yaks, a subspecies of bovid, are found throughout the Himalayas in both China and India and are commonly used for meat, fiber, and packing [12]. In the United States, yak hobby farms gained prominence in the mid-1990s where they are maintained as companion animals, bred for show, sold for their meat, and their hair used in fashion clothing [13]. These animals are polyestrous, seasonal breeders, and reach sexual maturity at approximately three years of age [14]. Yaks have been observed to have a variety of reproductive disorders including late maturity, long calving interval, and poor estrus expression [15]. Laparoscopic approaches could be applied to improve breeding soundness in yaks, selecting only the best genetics for reproduction similar to other species [8, 16]. Additionally, ovariectomy could be applied in yaks for reasons similar to cattle and other small ruminants, such as pregnancy prevention or treatment of ovarian disorders, including neoplasia, adhesions, and abscessation. The animal in the present study was electively sterilized as the owner expressed interest in maintaining the animal with the rest of the sexually mature herd with lack of interest in future reproduction.

Minimally invasive laparoscopic surgery has been applied increasingly in large animal surgery for ovariectomy, cryptorchidectomy, hernioplasty, and exploratory surgery [1, 17–20]. Ovariectomy or ovariohysterectomy is performed to prevent estrus, inappropriate lactation, aggressive behavior, fertility, and neoplasia of the reproductive tract [1, 20]. Although the required specialized surgical equipment required increased cost, minimally invasive approaches have been shown to reduce hospital stay, result in an earlier return to function, and reduce postoperative pain in both animals and humans [21–23]. Some benefits of a minimally invasive approach for ovary removal include smaller incisions that require less time to heal; less traction on the ovarian pedicle, which reduces postoperative pain; and complete observation of the operative field [17, 24]. A unique benefit of laparoscopic surgery in livestock species is the ability for earlier return to previous routine—one that does not typically include stall confinement or a regimen that involves daily management. Due to smaller incisions that result in less postoperative pain and a reduced risk of incisional complications, these animals require less intensive management at home following surgery relative to confinement and necessary medications. The yak in the present report received a single dose of anti-inflammatory medication to control postoperative pain, and the animal was discharged within 48 hours following surgery. We believe this was due to the minimally invasive approach used and further estimate that application of a traditional open approach would have resulted in a higher likelihood that the animal would require additional treatment and monitoring at home.

This is the first known report of laparoscopic ovariectomy being performed in a yak. The minimally invasive technique was found to be similarly easy to perform as in other small ruminant species, and clinicians should feel confident to perform the procedure in a yak if they have experience of such approach in other larger animal species. No perioperative complications were encountered, and at 12 months following surgery, the animal was reported as healthy, and the owner was highly satisfied with the outcome.
